# Volcanic textures control the mechanical behaviour of Mesoamerican obsidians

**DOI:** 10.1098/rsos.261210

**Published:** 2026-10-14

**Authors:** Luiz Pereira, Geovana L. Santana, Qiuju Zheng, Ricardo F. Lancelotti, Augusta Isaac, Alejandro Pastrana, Hugo Delgado-Granados, Edgar D. Zanotto, Donald B. Dingwell

**Affiliations:** ^1^ GEOLAB, Hangzhou International Innovation Institute, Beihang University, Hangzhou, China; ^2^ Department of Earth and Environmental Sciences, Ludwig-Maximilians-Universität München, Munich, Germany; ^3^ High-Temperature Materials Laboratory (HTML) – (Brazil-China Joint Laboratory); ^4^ Department of Materials Engineering, Federal University of São Carlos, São Carlos, Brazil; ^5^ School of Materials Science and Engineering, Qilu University of Technology, Jinan, People's Republic of China; ^6^ Metallurgical and Materials Engineering Department, Federal University of Minas Gerais, Belo Horizonte, Brazil; ^7^ Dirección de Estudios Arqueológicos, Instituto Nacional de Antropología e Historia, Mexico City, Mexico; ^8^ Instituto de Geofísica, Universidad Nacional Autónoma de México, Mexico City, Mexico; ^9^ State Key Laboratory of Silicate Materials for Architectures (SMART), Wuhan University of Technology, Wuhan, People's Republic of China

**Keywords:** glass, Aztecs, Teotihuacan, mechanical properties, archaeology

## Abstract

Obsidian—natural volcanic glass—was exploited for millennia and played a central role in the technological and socio-economic development of pre-Hispanic societies across Mesoamerica. The selection of obsidian from specific deposits has been linked to differences in visual appearance and patterns of use; however, the material properties underlying these preferences remain poorly understood. Here, we investigate obsidians from the Sierra de Las Navajas deposit—the most exploited obsidian deposit in Mesoamerica. We combine ball-on-three-ball measurements to quantify fracture strength with nanoindentation to quantify hardness, complemented by X-ray tomography and scanning electron microscopy. Mechanical behaviour varies among obsidian families and sampling locations and is primarily controlled by textural characteristics, notably the size and morphology of inclusions. These textures reflect distinct volcanic processes that govern material performance. Our results establish the first quantitative framework linking volcanic textures and mechanical performance in obsidians from Sierra de Las Navajas, providing a physical basis for evaluating how geological processes may have influenced obsidian utilization in pre-Hispanic Mesoamerica.

## 1. Introduction

More than two centuries ago, during his travels through the Viceroyalty of New Spain (present-day Mexico), Alexander von Humboldt described the famous obsidian mines of the Sierra de las Navajas, known locally as the ‘Mountain of Knives’, highlighting the importance of these deposits for pre-Hispanic societies [1]. These obsidian-rich regions of central Mexico were occupied and exploited by several major pre-Hispanic civilizations, including the Teotihuacan, Toltec and later the Mexica (Aztecs) [2–5]. Their principal urban centres were located relatively close to major obsidian deposits of Sierra de Las Navajas, within approximately 50–150 km. These societies established extensive extraction, manufacturing and trade networks centred on obsidian resources, which played a fundamental role in domestic activities, warfare, religion and long-distance exchange throughout Mesoamerica. Obsidian was extensively used for the manufacture of blades, cutting tools and weapons throughout Mesoamerica, whereas visually distinctive varieties were often selected for ornamental and ritual artefacts [3]. These materials were extensively used for the manufacture of prismatic blades, knives, projectile points, scrapers, drills and other cutting objects, whereas visually distinctive varieties were often selected for ornamental and ritual artefacts. Among these technologies, the production of prismatic blades represented one of the most sophisticated lithic industries in pre-Hispanic Mesoamerica. This manufacturing process relied on the controlled propagation of conchoidal fractures to detach long, regular blades from prepared cores, requiring obsidian with high mechanical quality and predictable fracture behaviour.

The Trans-Mexican Volcanic Belt (TMVB) is an approximately 1000 km long volcanic arc generated by the subduction of the Rivera and Cocos plates beneath the North American plate [6,7]. Several volcanic centres within the TMVB erupted obsidian, including Las Navajas volcano and Tequila volcano in the western sector [8–11]. In the eastern TMVB, the Sierra de Las Navajas volcano was constructed between ≈ 2.5 and 2.2 Ma and produced large volumes of obsidian [12]. This deposit constitutes one of the most important obsidian sources in Mesoamerica [3–5] and contains obsidians displaying distinct colours and internal textures [13].

Obsidian can be defined as silica-rich to intermediate volcanic material dominated by a glassy matrix with only minor crystallization [14–17]. Contrary to the common assumption that glass forms solely through extremely rapid cooling, these melts can in fact transform to the glassy state over long durations, demonstrating an exceptionally high glass-forming ability [15,16]. Obsidian occurs across a wide spectrum of volcanic settings, ranging from pyroclastic deposits to effusive lava flows, and spans a broad range of glass chemistries, from calc-alkaline and peralkaline rhyolites to more intermediate compositions such as andesites, trachytes and phonolites [13–20]. The ones generally found in central Mesoamerica are typically rhyolitic and peralkaline [13,21,22]. These materials are often characterized by a low but highly variable content of crystals and vesicles.

The mechanical behaviour of brittle materials, such as glasses, is strongly influenced by such internal heterogeneities [23–28]. Vesicles and crystals may act as stress amplifiers and therefore control fracture initiation and propagation, and finally thus, strength variability and failure probability [24–28]. Consequently, the abundance, size, distribution and morphology of these secondary phases are expected to generate significant differences in mechanical performance of brittle materials, such as glasses. Because obsidian develops exceptionally sharp and predictable conchoidal fractures, it became the preferred material for the manufacture of cutting tools and weapons in civilizations in Mesoamerica, before the Spanish invasion [3–5].

Despite extensive geological and archaeological investigations, quantitative relationships between volcanic microstructures and the mechanical performance of different obsidian varieties remain poorly constrained. As a result, it remains unclear whether the preferential selection of specific obsidian resources by pre-Hispanic societies was associated with measurable differences in material performance. We therefore hypothesize that the contrasting volcanic textures observed among the Sierra de Las Navajas obsidian varieties produce distinct mechanical behaviours. Specifically, obsidians containing abundant vesicles and/or crystalline inclusions are expected to exhibit lower fracture strength and less predictable fracture behaviour owing to a larger population of critical flaws, whereas texturally homogeneous obsidians should display higher mechanical reliability and more regular conchoidal fracture.

In this contribution, we investigate the mechanical behaviour of the principal obsidian varieties from the Sierra de Las Navajas deposit and evaluate whether their mechanical performance can be related to volcanic textures. The Sierra de Las Navajas deposit contains several visually and texturally distinct obsidian varieties. We focus on three representative families: vesicle-bearing obsidians (VBO), characterized by abundant vesicles and locally banded textures, characterized by a golden appearance; crystal-bearing obsidians (CBO), containing few dispersed microcrystals within a dominantly glassy matrix, characterized by a black appearance; and homogeneous dark obsidians (HDO), characterized by an almost complete absence of crystals and vesicles, and also characterized by black appearance (figure 1). Using these materials, we combine ball-on-three-ball (B3B) fracture-strength measurements with nanoindentation as well as X-ray tomography and scanning electron microscopy (SEM). By establishing quantitative relationships between microstructure and mechanical performance, we provide new insights into how geological processes generated functionally distinct obsidian resources that were selectively exploited by pre-Hispanic societies.

**Figure 1.**
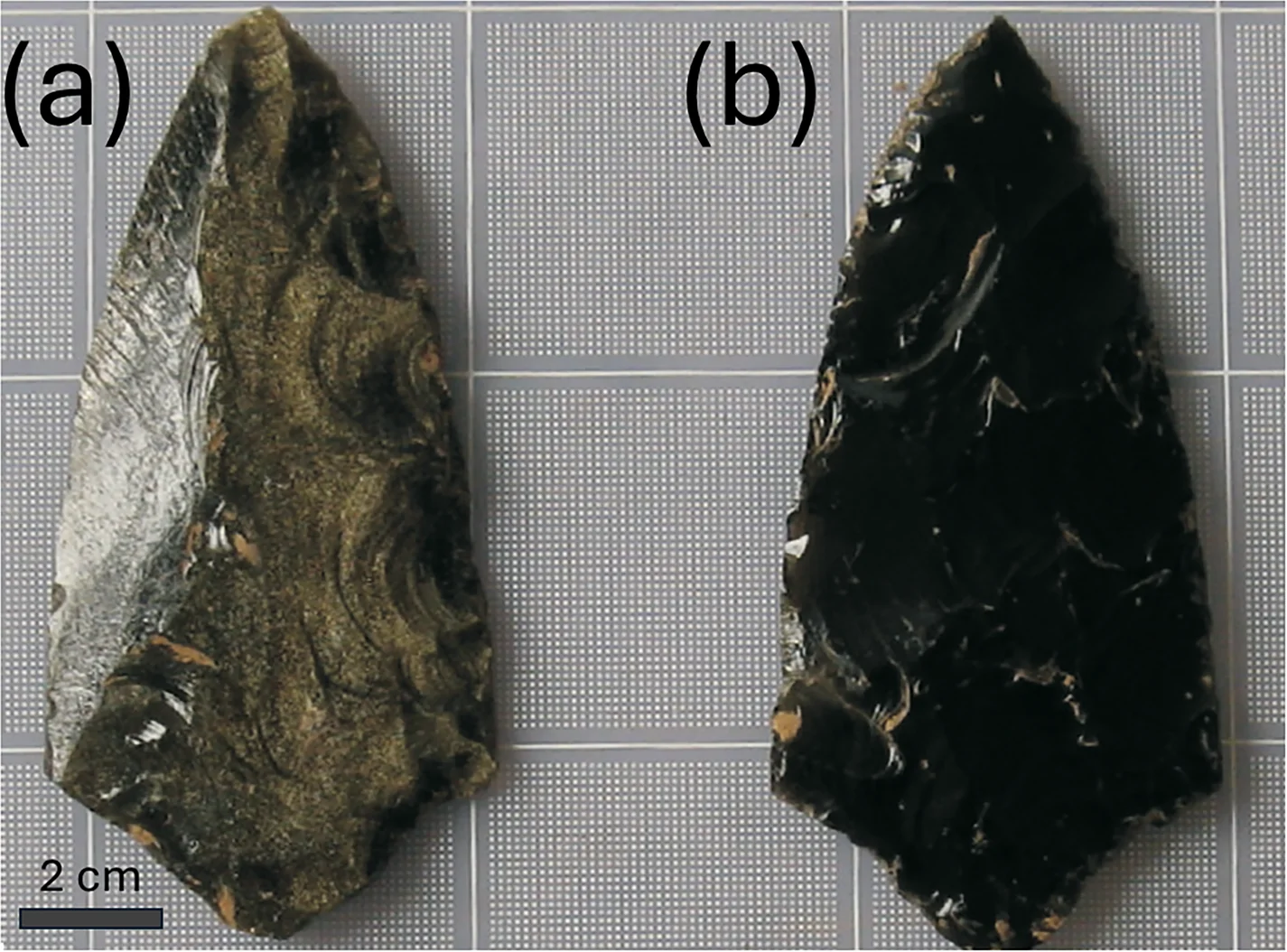
Photographs of the dorsal surface of two obsidian dart-point preforms excavated by Dr Alejandro Pastrana from the Sierra de Las Navajas archaeological obsidian source (Hidalgo, Mexico). Panel (a) shows a preform manufactured from golden, vesicle-bearing obsidian (VBO), whereas panel (b) shows a preform manufactured from homogeneous dark obsidian (HDO). These artefacts illustrate the use of different obsidian varieties in the manufacture of projectile-point technology in pre-Hispanic Mesoamerica.

## 2. Materials and methods

### 2.1. Sampling obsidians

Obsidian samples were collected from the Sierra de Las Navajas deposit (Hidalgo, Mexico). Three distinct obsidian families were investigated in this study: (i) vesicle-bearing obsidians, (ii) crystal-bearing obsidians, and (iii) homogeneous dark obsidians. The present study focuses on specimens collected from three sectors of the Sierra de Las Navajas deposit: quadrant 4E (samples HDO-1 and HDO-2), quadrant 8B (sample CBO-1) and quadrant 5C (samples VBO-S and VBO-E). A detailed map of the deposit, including the sampling quadrants and their locations, is available in the original publication [5]. The suffixes S and E for VBO-S and VBO-E denote samples containing predominantly spherical and elongated vesicles, respectively.

### 2.2. Chemical composition

Chemical composition was obtained by energy-dispersive X-ray spectroscopy (EDS) coupled to an SEM instrument (FEI Inspect S50, FEI Company) equipped with a Bruker X-ray detector. SEM–EDS was selected owing to its ability to provide spatially resolved compositional information coupled with direct microstructural imaging, enabling us to identify and select areas of glassy matrices and secondary phases independently. Prior to analysis, the samples were polished to obtain flat and representative surfaces. EDS measurements were performed under high-vacuum conditions using an accelerating voltage of 20 kV and a working distance of 10.4 mm. For each sample, the analysis was carried out over relatively large selected surface areas (typically of the order of 10^4^ µm^2^) at 500× magnification, in order to obtain representative average compositions of the glass matrix rather than localized spot analyses. Each acquisition was performed for 6 minutes. The elemental compositions obtained by EDS were converted into oxide concentrations using stoichiometric factors. In samples containing heterogeneous crystalline inclusions, additional area-selected EDS analyses were performed directly on these regions to evaluate their local composition.

### 2.3. Hydroxyl content

Fourier transform infrared (FTIR) spectra of the glass samples were acquired using a Nicolet iS10 spectrometer (Thermo Fisher Scientific, Madison) at a resolution of 4 cm^–1^ at 12 different positions for the same sample. Each scan was performed over the range 2000–4000 cm^−1^ with a step size of 2 cm^‒1^. Background correction of air (from 32 scans) was applied to each sample scan, and the final spectrum was averaged from 16 scans. The hydroxyl content in the glass is directly proportional to the IR absorbance per unit sample thickness at approximately 3670 cm^‒1^, following Beer's law. The two-point tangent method (at 2600 and 3800 cm^-1^) was used to establish a baseline for each spectrum, which was corrected to determine the actual hydroxyl IR absorbance at 3670 cm^‒1^ (Abs_3670_ cm^‒1^). According to Beer's law, the hydroxyl content (ppm OH by weight) was calculated using the hydroxyl IR extinction coefficient (77 l mol^‒1^ cm^‒1^) near the reported wavenumber for silicate glasses. The spectra of the investigated materials are presented in supplemntary material, figure S1.

### 2.4. X-ray tomography observation

We performed high-resolution X-ray computed tomography using a ZEISS Xradia Versa 610 system, enabling full three-dimensional characterization of the specimen. This three-dimensional X-ray microscope enables non-destructive imaging of internal structures with high spatial resolution. The scans were carried out under the following conditions: an acceleration voltage of 120 kV, a current of 146 µA and an exposure time of 1.5 s per projection. A total of 2034 radiographic projections were acquired, resulting in an isotropic voxel size of 8.50 µm. The distance from the X-ray source to the detector (detector-to-rotation axis distance) was approximately 109.8 mm, and the distance from the source to the rotation axis (source-to-rotation axis) was approximately 36.5 mm. The optical magnification used was 0.396×. The raw images had a resolution of 2048 × 2008 pixels with a 16-bit depth (short data type). Volume reconstruction was performed using XM Reconstructor, the software provided by ZEISS. Subsequent image processing and quantitative analysis were conducted using Avizo 2024.1 (Thermo Fisher Scientific). Image processing steps included phase segmentation, pore extraction and morphological quantification of the features of interest.

To characterize the size and shape of the pores segmented from the tomographic volumes, two geometric descriptors were extracted using Avizo software (Thermo Fisher Scientific): Length3D and FeretShape3d. The former corresponds to the maximum extension of each pore along its principal axis. It is computed by fitting an equivalent ellipsoid to the pore volume and determining the largest semi-axis of that ellipsoid. This metric provides a robust estimation of the object's longest dimension in three-dimensional space. The latter is derived from the Feret diameters, which represent calliper-like measurements of the object's size along multiple orientations. In three dimensions, the software calculates Feret diameters along the principal directions of the pore and reports a shape descriptor defined as the ratio between the maximum and minimum Feret diameters. This ratio reflects the degree of elongation or flatness of the pore, with higher values indicating more anisotropic shapes.

### 2.5. Fracture strength using ball-on-three-ball method

The five different obsidian sets were cut into discs with a diameter of 14 ± 0.2 mm and a thickness of 1.2 ± 0.1 mm, and polished on parallel faces to optical quality. To avoid the influence of surface flaws on the measured results, all samples were subjected to an identical polishing procedure. This approach ensured that polishing did not introduce an additional variable that could affect the failure behaviour.

Fracture strength measurements were performed on the polished obsidian discs using B3B tests. The experiments were conducted using an MTS Criterion^TM^ Model 43 system on 30 specimens per obsidian type, totalling 150 discs, with continuous acquisition of stress–displacement data [29,30]. Fracture strength was defined as the stress at which the load dropped abruptly in the stress versus displacement plot, marking the mechanical failure of the sample. The flexural strength results were evaluated using a Weibull statistical analysis, which is the standard framework for capturing the probabilistic nature of fracture in brittle solids [27,28]. This approach allows the extraction of both the characteristic failure strength (average value) and the reliability of the material, expressed through the Weibull modulus (*m*).

### 2.6. Hardness using nanoindentation method

Hardness of glass surfaces was analysed with a nanoindenter (Agilent G200, Keysight, USA) using a Berkovich tip in the continuous stiffness measurement mode. The indentation depth was set to 2000 nm. A polycrystalline pure aluminium standard was used as a reference material to calibrate the tip area function. Since the glass samples are heterogeneous, around 15 indentations were made on each local region with specific textural features to ensure repeatability. In the local region, every two indentations have an interval of 20 µm, and the indentations in the horizontal and vertical directions form a cross shape.

## 3. Results

### 3.1. Textural features

Figure 2 presents representative photographs and cross-sectional images extracted from the X-ray computed tomographic volumes. They highlight the visual and textural features of the investigated obsidian samples. Clear differences are observed among the samples, particularly in the presence, abundance, size and morphology of secondary phases, more precisely, vesicles. In addition to these textural observations, we also report the bulk chemical compositions of the samples in terms of their major oxide contents (supplementary material, table S1).

**Figure 2.**
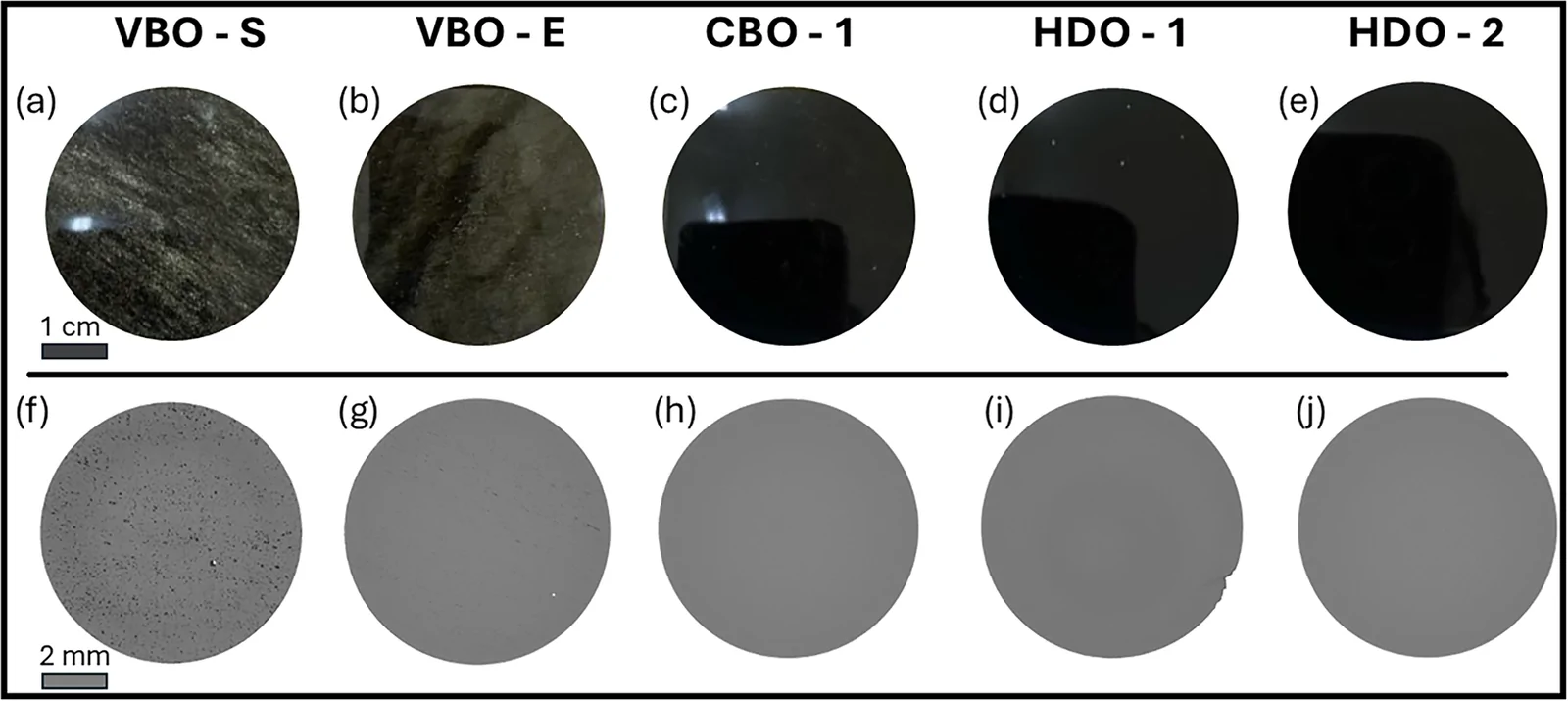
Photographs and representative cross-sectional images extracted from X-ray computed tomographic volumes of obsidian samples from the Sierra de Las Navajas deposit (Hidalgo, Mexico). Panels (a–e) show photographs of the analysed materials: (a) vesicle-bearing obsidian with spherical vesicles (VBO-S), (b) vesicle-bearing obsidian with elongated vesicles (VBO-E), (c) crystal-bearing obsidian (CBO-1), (d) homogeneous dark obsidian (HDO-1) and (e) homogeneous dark obsidian (HDO-2). Panels (f–j) present the corresponding cross-sectional slices extracted from the X-ray computed tomographic datasets of samples (a–e), respectively. Panels (f–j) reveal the contrasting internal textures of the investigated materials, in which dark regions correspond to low-density phases, primarily gaseous inclusions (vesicles), whereas the grey matrix represents the glassy phase. Crystalline phases, when present, appear as brighter features owing to their higher X-ray attenuation.

The localized heterogeneous regions corresponding to vesicle fillings were identified in the vesicle-bearing obsidian samples (VBO-S and VBO-E), as already suggested by the textural contrast observed in figure 2. These regions are illustrated in figure 3 by representative SEM micrographs of these vesicle-filling regions. These regions were further investigated by EDS area analyses performed over selected heterogeneous domains, and the corresponding compositions are summarized in table S2 in the supplementary material. The analyses revealed significant enrichment in BaO and Cr_2_O_3_, reaching approximately 16 and 4 mol% in VBO-S, and 9 and 2 mol% in VBO-E, respectively, accompanied by lower SiO_2_ contents (approx. 64–67 mol%) compared to the surrounding glassy matrix (approx. 79 mol%).

**Figure 3.**
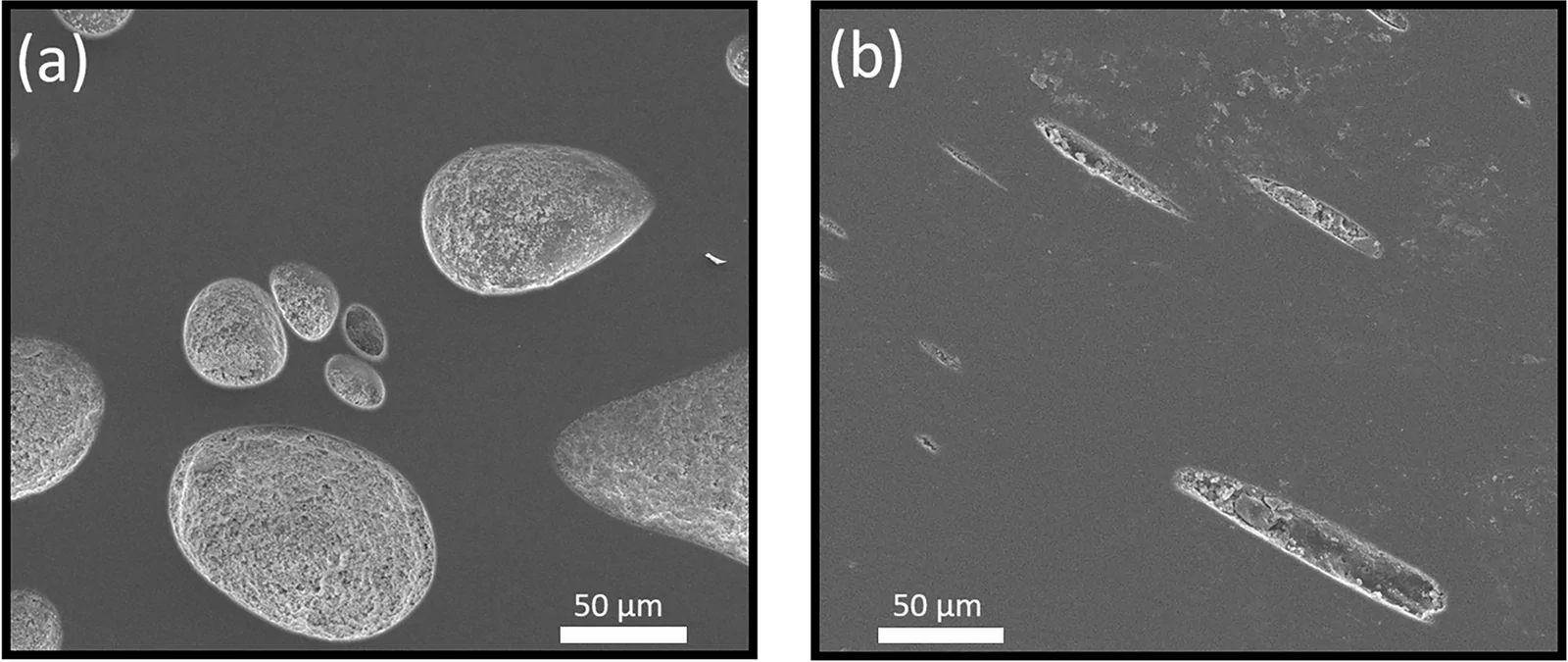
Localized vesicle-filling regions identified in vesicle-bearing obsidians (VBO-S and VBO-E). Representative SEM micrographs illustrating the morphology and spatial distribution of impurity-rich regions embedded within the glassy matrix and associated with Si–Na–Ba–Cr-rich inclusions. (a) VBO-S displays predominantly rounded impurity-rich inclusions, whereas (b) VBO-E is characterized by elongated impurity-rich inclusions.

To further elucidate these textural differences, figure 4 combines X-ray computed tomographic images and their corresponding quantitative analyses for the two samples. These chosen samples are the ones exhibiting the largest sizes and highest volume fractions of inclusions (i.e. VBO-S and VBO-E). The tomographic data reveal the three-dimensional assessment of vesicle size and morphology. The Length3D distributions reflect the volumetric size variation of the vesicles, whereas the FeretShape3D parameter quantifies their shape anisotropy derived from Feret-diameter analysis. The combined examination reveals marked differences in aspect ratio and morphological heterogeneity between the samples. More specifically, figure 4a–d illustrates the textural characteristics of VBO-S, wherein figure 4a contains a representative cross-sectional slice from the VBO-S X-ray tomographic volume, while figure 4b presents the X-ray tomographic rendering that resolves the three-dimensional vesicle population across the entire specimen. Figure 4c displays the corresponding bubble-size distribution histogram, and finally figure 4d presents the three-dimensional bubble-shape distribution. Figure 4e–h presents the equivalent dataset for sample VBO-E. As can be seen in figure 4, one might find larger vesicles and more elongated ones for this latter sample.

**Figure 4.**
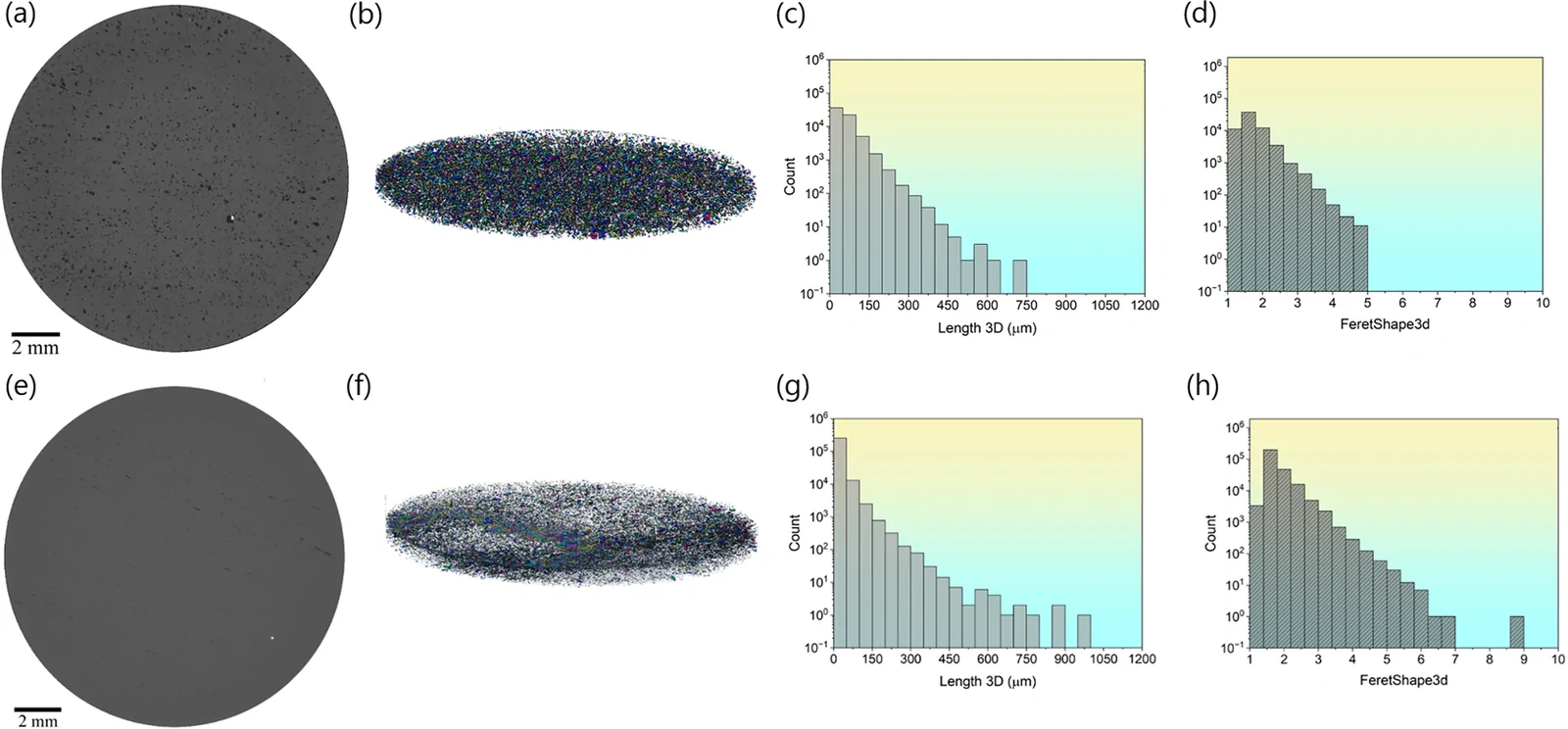
Textural features of the two obsidian samples with the highest bubble fractions (VBO-S and VBO-E) obtained by X-ray computed tomography. Panels show complementary textural information for each sample: (a) representative cross-sectional slice from the VBO-S X-ray computed tomographic volume, (b) three-dimensional rendering of the sample volume showing the bubble network in VBO-S highlighted in colour and the glassy matrix rendered in transparent blue, (c) bubble-size distribution histogram for VBO-S, (d) three-dimensional vesicle-shape distribution based on Feret-diameter analysis for VBO-S, (e) representative cross-sectional slice from the VBO-E volume, (f) X-ray computed tomography rendering highlighting the three-dimensional bubble network in VBO-E, (g) bubble-size distribution histogram for VBO-E and (h) three-dimensional bubble-shape distribution based on Feret-diameter analysis for VBO-E.

### 3.2. Nano- to macroscale mechanical responses

From a mechanical perspective, the pronounced microstructural variations observed among the samples in figures 2–4 may imply corresponding differences in the distribution and density of internal ‘flaws’, and therefore mechanical behaviour [24,26]. To quantify this variability, we performed mechanical characterization from nanoscale to macroscale. Figure 5 presents the nano-hardness (*H_n_*) measured for the investigated obsidian samples. Measurements were performed at two distinct locations in each sample to assess spatial homogeneity. The insets of figure 5 show optical micrographs acquired at the exact locations of the nanoindentation measurements, highlighting contrasts in the local textures of the investigated samples. Moreover, these optical micrographs indicate the precise indentation sites where the mechanical measurements were performed. A direct relationship can be observed between the measured nano-hardness values and the textural features at which the measurements were performed. Samples VBO-E, CBO-1, HDO-1 and HDO-2 exhibit nearly constant nano-hardness values, both within individual samples and across this group of obsidians. By contrast, the VBO-S sample exhibits systematically lower nano-hardness values that are strongly dependent on measurement position, reflecting spatial heterogeneities within the material. The higher vesicle fraction for this latter sample increases the likelihood that the indentation volume is affected by nearby vesicles, as illustrated in the inset micrographs.

**Figure 5.**
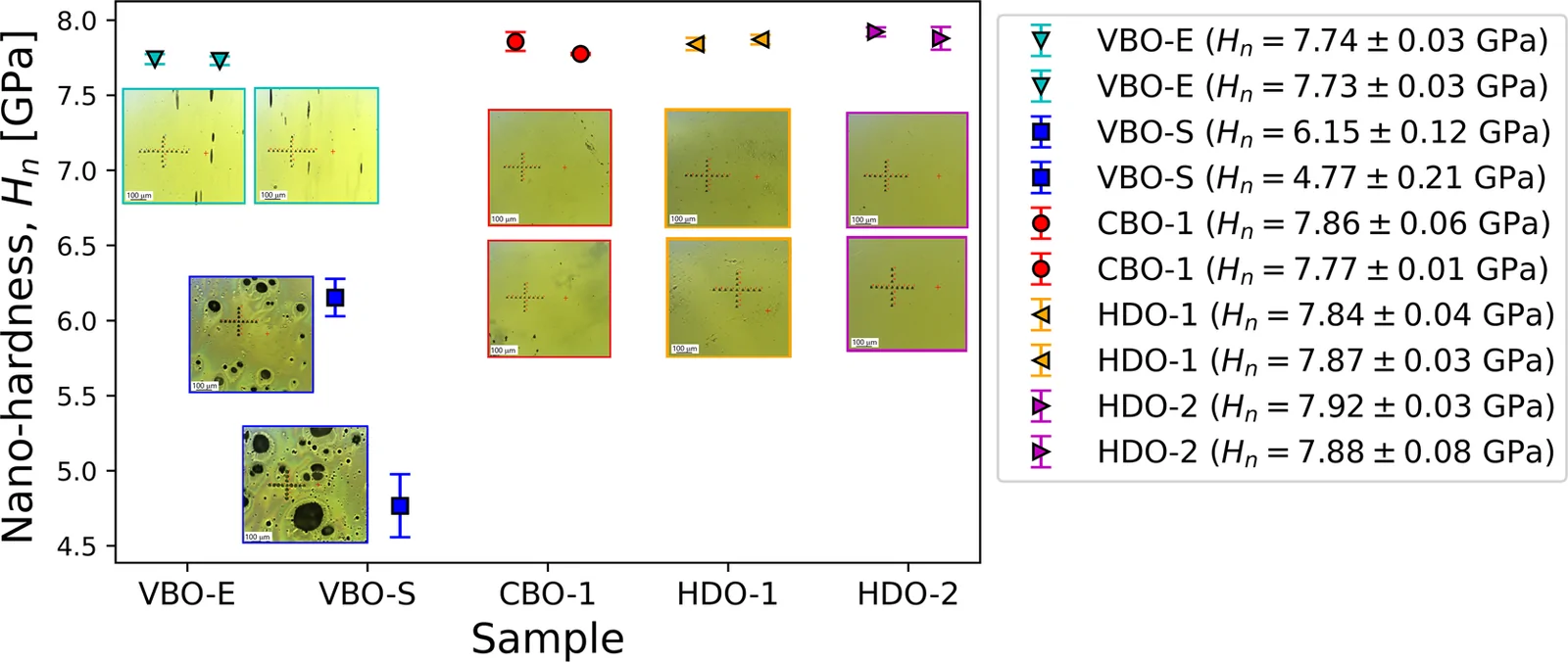
Nano-hardness of obsidians from Sierra de Las Navajas deposit (Hidalgo, Mexico). The plot displays the measured nano-hardness values by the nanoindentation technique for the investigated obsidian types: vesicle-bearing obsidian with elongated vesicles (VBO-E), vesicle-bearing obsidian with spherical vesicles (VBO-S), microcrystal-bearing obsidian (CBO-1) and homogeneous dark obsidians (HDO-1 and HDO-2). Symbols represent the average value of each dataset, and error bars correspond to ±1*σ.* Insets show optical micrographs taken at the nanoindentation sites, the crosses are the nano-hardness measurement points and the optical micrographs are framed in the same colour as the corresponding data symbols.

Biaxial flexural strength was determined using B3B fracture tests. By contrast with nanoindentation, B3B testing probes bulk material volumes and therefore captures the influence of the glassy matrix together with internal flaws and microstructural heterogeneities on fracture behaviour at the macroscale. Figure 6 displays the B3B test results of flexural strength using a Weibull plot. The Weibull equation, in its linear form (equation (3.1)), displays the double natural logarithm of 1/(1 − *P_f_*), where *P_f_* is the failure probability, as a linear function of the natural logarithm of the flexural strength σ. In this representation, the slope corresponds to the Weibull modulus (*m*) and the intercept is related to the characteristic strength (σ_0_) and to the Weibull modulus (m).

(3.1) $$\ln\left[\ln\left(\frac{1}{1-P_{f}}\right)\right]=mln\left(\sigma \right)-mln\left(\sigma_{0}\right).$$

**Figure 6.**
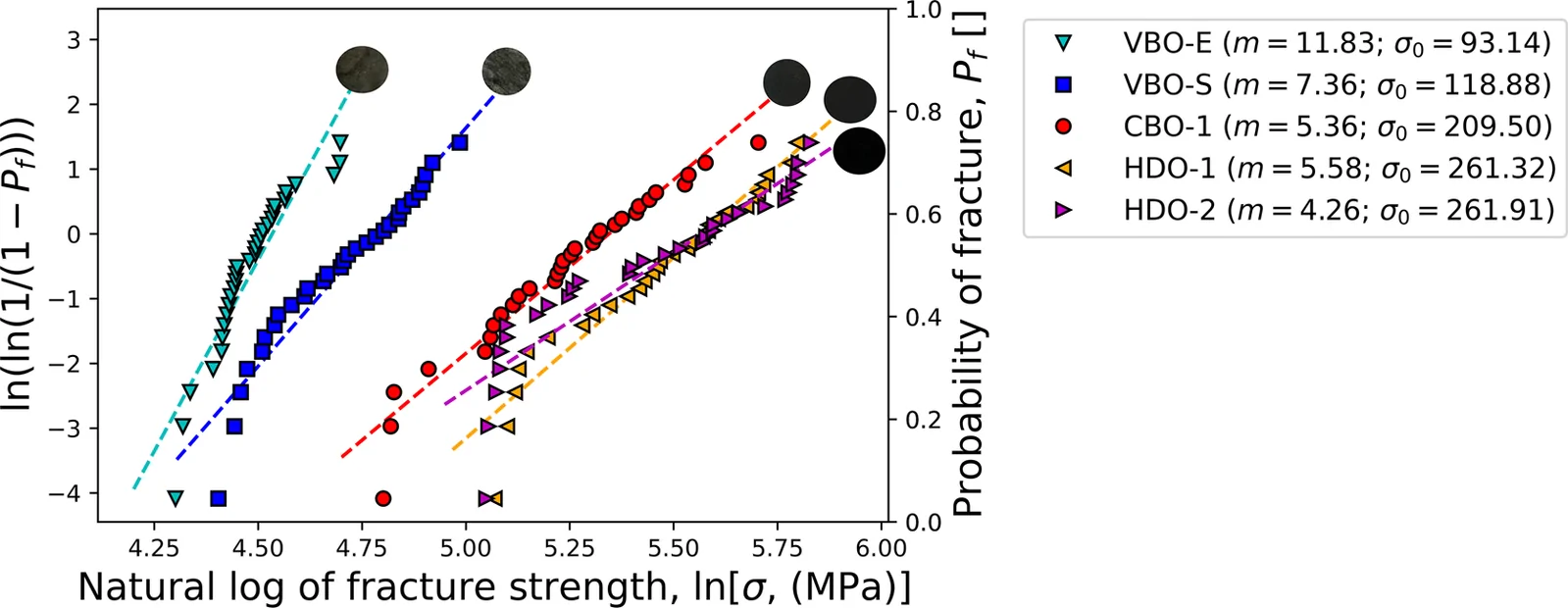
Weibull analysis of flexural strength for the studied obsidian samples from the Sierra de Las Navajas deposit (Hidalgo, Mexico). The Weibull plot displays the double natural logarithm of one divided by one minus the failure probability as a function of the natural logarithm of fracture strength, revealing clear mechanical distinctions among the samples. The legend identifies each dataset using the adopted sample nomenclature, where VBO refers to vesicle-bearing obsidians, with E and S indicating elongated and spherical bubble morphologies, respectively, CBO refers to microcrystal-bearing obsidians, and HDO denotes homogeneous dark obsidians. The corresponding Weibull modulus for each of the studied samples is also reported together with the characteristic strength values. Besides, the small circles at the top of the curves are small photos illustrating their different appearance linked to the presence of vesicles.

The B3B tests were conducted on 30 specimens, a sample size considered statistically robust for analysing the strength of brittle materials [26]. A secondary *y*-axis corresponding to the probability of fracture is also displayed. This plot reveals clear differences between the samples in terms of both their characteristic strength (average flexural strength value) and their ‘reliability’, which can be correlated to the Weibull modulus. This parameter reflects the width of the strength distribution: larger values of *m* indicate a narrower range of fracture-strength values, whereas lower values of *m* correspond to a wider range of strengths for failure. In other words, samples with higher *m* values are mechanically more reliable, whereas those with lower *m* values exhibit greater variability and are therefore less reliable, being able to undergo failure in a broader range of applied loads. Another key information from the Weibull plot is the characteristic strength, which can be obtained from the stress corresponding to a failure probability of 63.2%, which comes from the evaluation of the Weibull distribution at σ = σ_0_, where *P_f_* =  1 − *e*^−1^. As expected for a brittle material, the data vary linearly in the Weibull plot after the threshold region. Before this region, the probability of fracture rapidly approaches zero. This lower bound corresponds to a minimum applied stress below which failure is statistically improbable. All samples studied reveal such behaviour.

Fracture profile and the number of fragments after failure provide important information about the mechanical behaviour, the defect population controlling failure, as well as the elastic energy stored prior to failure. Figure 7a presents the distribution of the number of fragments for the investigated samples in the form of box plots, together with the optical micrographs illustrating samples after mechanical failure. Figure 7b shows the average number of fragments as a function of the characteristic strength, revealing a positive correlation between the two.

**Figure 7.**
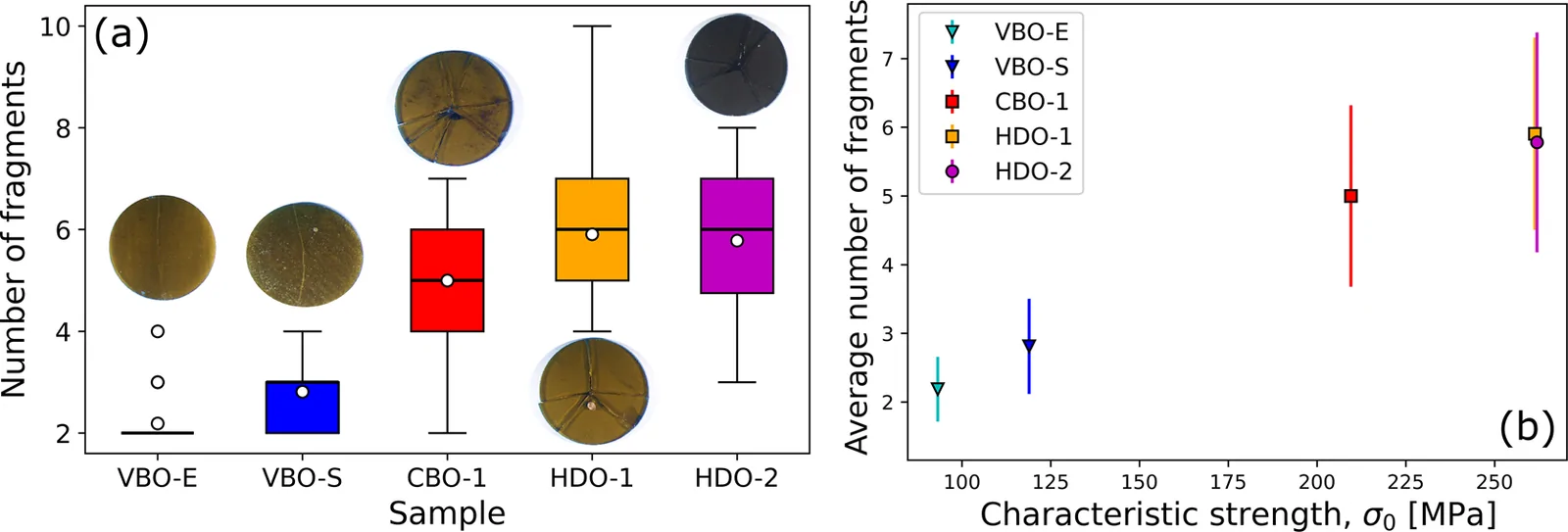
Box plots of the number of fragments and characteristic strength for the five studied samples from the Sierra de Las Navajas deposit (Hidalgo, Mexico). (a) The box plots illustrate the number of fragments for the five studied samples together with the optical micrographs illustrating the crack behaviour and (b) the average number of fragments as a function of the characteristic strength from the Weibull plot—the symbols represent the average value of each dataset, and error bars correspond to ±1*σ*.

## 4. Discussion

### 4.1. Nanoscale mechanical response

Nano-hardness results, together with the optical micrographs illustrating the locations of the measurements, provide relevant insights into the interplay between local mechanical behaviour and local texture in these samples. For instance, the sample containing elongated vesicles presents a relatively lower abundance, as evidenced by X-ray computed tomography analyses. This feature allowed nano-hardness measurements to be performed in regions that are free of vesicles (see figure 5 insets). As observed in the nano-hardness results for this sample, measurements performed at different locations show only minor variations, with nano-hardness remaining nearly constant at *H_n_* =  7.74  ± 0.03 GPa and *H_n_* =  7.73  ± 0.03 GPa and also showing a relatively low error bar, which is highlighting its locally homogeneous mechanical response. By contrast, the sample containing spherical vesicles exhibits a much higher vesicle abundance. Therefore, nano-hardness measurements in this sample were performed in close proximity to vesicles (see inset of figure 5). We therefore interpret the large variability observed in the nano-hardness data for this sample as a direct consequence of its pronounced textural heterogeneity associated with the high vesicle volume fraction as well as a broader vesicle size distribution (figure 5). This sample displays a markedly softer mechanical response, with nano-hardness values of *H_n_* =  6.15  ± 0.12 GPa and *H_n_* =  4.77  ± 0.21 GPa. In addition to the lower nano-hardness values, the relatively large error bars further highlight the pronounced heterogeneity of these obsidian samples at local scales. This heterogeneity also varies markedly within a single specimen, as evidenced by the differences observed between measurements performed at opposite locations of the same disc (centimetre scale). Nano-hardness in glasses depends on the hydroxyl content [31]. FTIR spectroscopy can be used to quantify the OH^−^ concentration at the surface of glassy materials, and an increase in this species is generally associated with a decrease in nano-hardness [31]. The softest sample (i.e. VBO-S) exhibited hydroxyl contents ranging from 0.145 to 0.275 wt%, whereas a harder sample (e.g. HDO-2) contains significantly higher hydroxyl contents, ranging from 0.343 to 0.377 wt% (supplementary material, figure S1 and table S1). This observation indicates that the reduced nano-hardness measured for VBO-S cannot be attributed to hydroxyl content. Instead, it suggests that the lower hardness of VBO-S is primarily related to the presence of soft vesicle-filling regions and associated microstructural heterogeneities in the vicinity of the indentation sites (see figure 4).

The sample containing a small fraction of crystals presents a more homogeneous texture when compared to the VBO-S sample, although the presence of crystals still introduces minor heterogeneities. The two measurements done on this material show nano-hardness values of *H_n_* =  7.86 ± 0.06 GPa and *H_n_* =  7.77  ± 0.01 GPa, indicating a largely consistent mechanical response at the local scale, as reflected by the small uncertainties, while revealing more pronounced variability at the centimetre scale when comparing the two evaluated regions, although much smaller when compared to the sample rich in spherical vesicles (VBO-S).

The two remaining samples investigated herein correspond to the homogeneous dark obsidians, which are characterized by a vitreous matrix and an almost complete absence of solid or gaseous inclusions (supplementary material, table S1; and figure 1). The nano-hardness values measured for these two samples, collected from the same location, are very similar and largely overlap within experimental uncertainty (figure 5). This behaviour indicates that, at the nanoscale, these materials exhibit a highly uniform mechanical response. Moreover, this mechanical behaviour at the nanoscale shows uniformity, which persists across multiple length scales: it persists from different indentation positions within the same region (microscale) to opposite sides of the same specimen (centimetre scale) and even between distinct samples collected from the same outcrop (metre scale), resulting in an overall nearly constant nano-hardness.

Glass hardness is known to depend on both chemical composition and thermal history through the fictive temperature [32]. However, recent work has shown that natural obsidians can be formed through cooling periods that range from months to years, depending on the emplacement conditions, while remaining sufficiently rapid to suppress crystallization [16]. Because these cooling times are substantially longer than those required for hardness to approach a plateau during structural relaxation [32], the influence of thermal history is expected to be minor in fully cooled natural obsidians cooled at these long durations. Consequently, the mechanical differences observed here are more likely to reflect variations in volcanic features, such as compositions, than differences in thermal history.

We aim to compare and discuss these novel nano-hardness results for naturally occurring silicate glasses with those for silicate and borosilicate glasses reported in the literature. Ma *et al.* [33] showed that the nano-hardness of medium-boron pharmaceutical glass is around 7.5  ± 0.1 GPa. He *et al.* [34] investigated the nano-hardness of low-boron pharmaceutical glasses and they range from 6.5 to 7.5  ± 0.1 GPa. Kaspar *et al.* [35] measured the nano-hardness of International Simple Glass, which is a model glass used in radioactive waste immobilization, and it falls in 7.6 ± 0.6 GPa. Except VBO-S, the nano-hardness of the other four obsidian samples has a narrow range of 7.7 to 7.9  ± 0.03 GPa, which is higher than that of pharmaceutical glasses and nuclear waste glasses, in general. Moreover, these naturally occurring materials present, for most of the cases, smaller uncertainties than the synthetic glasses. Although the obsidian samples are relatively heterogeneous, the nano-hardness of the glassy part is very uniform, with the exception of sample VBO-S.

### 4.2. Macroscale mechanical response

The macroscale mechanical response of glasses is controlled by the features of critical defects [24–26]. The Weibull analysis (figure 6) reveals marked differences among the investigated obsidians, with fracture strengths ranging from ln(σ) ≈  4.5 (σ ≈  90 MPa) to ln(σ) ≈  5.55 (σ  ≈  260 MPa) and Weibull moduli between *m* ≈ 4 and 12. Notably, samples with higher Weibull moduli exhibit lower characteristic strengths, indicating a narrower yet more critical flaw population governing failure. These variations correlate with textural heterogeneities, more precisely the critical defect size and shape, highlighting the strong influence of volcanic processing and lava emplacement on the mechanical reliability of natural glasses.

Among the investigated samples, VBO-E presents both the largest vesicles and the highest degree of vesicle elongation, as revealed by X-ray computed tomography and optical microscopy (figures 2–4). These elongated vesicle shapes in the obsidians found in the studied location likely originate from deformation during lava-flow emplacement [13]. In silicate melts under flow, vesicle shape results from the competition between viscous stresses, which tend to deform, and surface-tension forces, which act to restore a spherical shape [36–43]. Larger vesicles are more susceptible to deformation because the restoring effect of surface tension decreases with increasing vesicle radius, making viscous stresses comparatively more effective. Vesicle elongation may further promote bulk viscosity decrease, which might favour additional deformation during flow [37,42].

From a mechanical perspective, elongated vesicles act as highly efficient stress amplifiers [24–26]. According to classical fracture mechanics, stress amplification increases approximately with the square root of the flaw aspect ratio, making elongated vesicles particularly effective at promoting failure. Consequently, samples dominated by large and elongated vesicles, such as VBO-E, fail at comparatively low applied stresses and exhibit the lowest biaxial flexural strength among the investigated obsidians (figure 6). Fractographic observations (figure 7a, insets) further show that cracks preferentially propagate along elongated vesicles, suggesting that failure frequently initiates from similar defects. Consistent with this behaviour, this sample also exhibits the lowest number of fragments after fracture, indicating limited elastic energy stored prior to failure [44].

The sample VBO-S contains a larger vesicle volume fraction than VBO-E. However, mechanical failure in brittle materials is not governed by the total number or volume fraction of defects, but rather by the most critical flaws controlling stress amplification [24–26]. Consequently, despite its higher vesicle content, VBO-S exhibits greater flexural strength than VBO-E. This behaviour arises because the vesicles in VBO-S are predominantly spherical and therefore generate significantly lower stress concentrations than elongated defects. The predominance of spherical vesicles suggests emplacement conditions in which surface-tension forces remained sufficiently effective to limit vesicle deformation. Moreover, the relatively high Weibull modulus obtained for VBO-S indicates a comparatively homogeneous population of critical flaws. Nevertheless, its Weibull modulus remains lower than that of VBO-E, consistent with a broader distribution of flaw sizes and morphologies.

The sample CBO-1 contains only a minor fraction of crystals, and its macroscopic mechanical behaviour closely approaches that of the homogeneous dark obsidians (HDO-1 and HDO-2). Consequently, the lower abundance of vesicles and other large defects reduces the probability of encountering a critical flaw capable of generating sufficient stress amplification to initiate fracture. As a result, these samples exhibit higher fracture strengths, as reflected by the Weibull distributions (figure 6). At the same time, the relatively low Weibull moduli obtained for these samples (*m* ≈ 4.3−5.6) indicate greater specimen-to-specimen variability. This suggests that, although these obsidians are mechanically stronger, their flaw populations are less homogeneous at larger spatial scales. Nevertheless, the lower-bound strength defined by the Weibull distributions remains significantly higher than of the vesicle-bearing obsidians, confirming their superior resistance to fracture.

The box plot analysis reveals a positive correlation between the average number of fragments produced during the B3B tests and the characteristic strength (figure 7b). Within the present dataset, this also implies a negative correlation between fragment number and Weibull modulus, since characteristic strength and Weibull modulus are themselves inversely related. Fragmentation arises from the release of elastic strain energy stored prior to failure. Because this energy scales approximately with the square of the fracture strength ($\sigma_{f}^{2}$), stronger specimens are expected to generate a larger number of fragments upon fracture [44]. Additional variability may arise from residual stresses inherited from the cooling history of glasses; however, these materials generally form upon really slow cooling such that it might not be the key factor underpinning the mechanical behaviour.

Interestingly, when coupling the nanoscale mechanical results obtained by nanoindentation (figure 5) with the macroscopic fracture behaviour (figure 6), a clear contrast emerges that highlights the dominant role of defects in controlling failure. Most samples, with the exception of VBO-S, exhibit comparable nano-hardness values, indicating similar intrinsic resistance of the glassy matrix. However, their macroscopic mechanical responses differ substantially. These differences arise from variations in the abundance, size, morphology and spatial distribution of defects, which govern stress amplification and crack initiation and propagation at larger length scales. Together, these results demonstrate that while nano-hardness reflects the intrinsic mechanical resistance of the glass matrix, it is the characteristics of the defect population that ultimately dictate fracture strength and reliability.

The present biaxial flexural-strength measurements combined with Weibull statistical analysis provide new quantitative insights into the mechanical reliability of natural obsidians. Although the investigated samples exhibit relatively similar intrinsic matrix properties at the nanoscale, their macroscopic mechanical behaviour varies considerably owing to differences in defect populations inherited from volcanic emplacement processes. In particular, vesicle morphology emerges as a first-order control on fracture behaviour, with elongated vesicles acting as highly efficient stress concentrators that substantially reduce fracture strength. More broadly, the results demonstrate that volcanic processes influence not only the texture of obsidians but also their mechanical reliability, establishing a direct link between lava-flow dynamics, microstructure development and the engineering-scale performance of natural volcanic glasses.

## 5. Implications

The results presented here demonstrate that volcanic textures exert a primary control on the mechanical performance of obsidians from Sierra de Las Navajas, providing a physical framework for evaluating their utilization by pre-Hispanic societies. By combining microstructural characterization with nano- and macroscopic mechanical analyses, we show that mechanically superior obsidians are characterized by a lower probability of containing critical flaws capable of initiating and propagating fracture. These materials exhibit higher fracture strengths and more predictable fracture behaviour because defects, when present, are generally smaller and less effective as stress amplifiers. Conversely, obsidians containing abundant vesicles, particularly large and/or elongated ones, exhibit reduced fracture strength owing to enhanced local stress amplification around critical defects. These observations are consistent with the hypothesis proposed at the beginning of this study (Introduction). The results demonstrate that textural heterogeneity inherited from volcanic processes translates directly into mechanical heterogeneity, whereas obsidians with more homogeneous textures exhibit greater fracture strength and more predictable fracture behaviour, properties that would have favoured their selection for the manufacture of cutting tools and weapons.

The homogeneous dark obsidians (HDO-1 and HDO-2) provide a clear example of this relationship. These materials contain very few inclusions and exhibit the highest fracture strengths among the investigated samples. Their relatively homogeneous microstructure promotes regular conchoidal fracture and predictable fracture behaviour during percussion and pressure knapping, properties highly desirable for the manufacture of cutting tools and weapons. Such properties are particularly advantageous for the manufacture of prismatic blades, where controlled crack propagation is essential to detach long, regular flakes from prepared cores while minimizing unintended fracture. The present results therefore provide a mechanical explanation for why certain obsidian varieties from Sierra de Las Navajas may have been preferentially selected for this highly specialized technology. In addition, their high fracture strength increases resistance to mechanical failure during use. By contrast, the vesicle-bearing obsidians (VBO-S and VBO-E) exhibit lower fracture strengths owing to the presence of abundant vesicles that act as stress amplifiers. In particular, elongated vesicles strongly amplify local stresses and promote premature failure. Although these materials are mechanically less suitable for applications requiring reliable edge formation and high resistance to fracture, they possess visually distinctive textures and optical effects that differentiate them from the more homogeneous varieties.

Taken together, these results establish a direct interplay between volcanic processes, obsidian textures and their mechanical performance. The textures observed in the investigated materials, including variations in vesicle abundance, size and morphology, constitute a geological record of the volcanic processes responsible for obsidian formation. These processes generated the microstructural heterogeneities preserved within the glass, which in turn determined the nature and distribution of critical flaws and thereby controlled fracture behaviour and mechanical performance. The correspondence between the measured mechanical properties and archaeological use patterns suggests that these volcanically generated textures played an important role in determining the technological suitability of different obsidian varieties. Consequently, this study provides a physical framework for understanding obsidian selection in pre-Hispanic Mesoamerica. More broadly, our findings indicate that the technological value of obsidian resources was ultimately rooted in the volcanic processes that generated them.

## 6. Conclusion

Volcanic textures exert a primary control on the mechanical performance of obsidians from Sierra de Las Navajas. Variations in vesicle abundance, size and morphology generate distinct populations of critical flaws that govern fracture behaviour and strength, whereas the intrinsic hardness of the glass matrix remains largely invariant among most of the investigated obsidians. These findings demonstrate that the mechanical performance of obsidian is ultimately governed by the volcanic processes responsible for its formation. By controlling the development of textural heterogeneities and critical flaw populations, these processes determine the fracture behaviour and strength of the resulting materials. Consequently, this study establishes a direct link between volcanic processes, obsidian textures and mechanical performance, providing the first quantitative physical framework for investigating obsidian selection in pre-Hispanic Mesoamerica. More broadly, our results offer archaeologists a mechanistic basis for interpreting patterns of material choice and assessing how the geological evolution of obsidian deposits may have influenced the technological value of the resources they provided.

## Supplementary Material



## Data Availability

The datasets and Python script supporting this study have been deposited in the Zenodo repository and are available at [45]. Supplementary material is available online [46].
