# Supplementary material for: Volcanic textures control the mechanical behaviour of Mesoamerican obsidians
Source: R Soc Open Sci. 2026 Oct 14;13(10):261210. doi: 10.1098/rsos.261210 (PMC13682573; doi:10.1098/rsos.261210)
Supplement: Supplementary file 1 [file rsos.261210.zip › rsos.261210/rsos261210_si_002.docx]

**Table S1. Major oxide composition (mol%) of the investigated obsidian samples from the Sierra de las Navajas deposit (Hidalgo, Mexico), determined by energy-dispersive X-ray spectroscopy (EDS) coupled with scanning electron microscopy (SEM).** **The range of OH^-^ contents (ppm by wt.%), determined by FTIR spectroscopy, is also reported.**

| *Oxides* | *VBO-E* | *VBO-S* | *CBO-1* | *HDO-1* | *HDO-2* |
| --- | --- | --- | --- | --- | --- |
| SiO_2_ | 79.4 ± 3.0 | 79.0 ± 3.1 | 79.0 ± 2.9 | 79.9 ± 2.6 | 79.4 ± 3.1 |
| TiO_2_ | 0.2 ± 0.1 | 0.1 ± 0.0 | 0.2 ± 0.1 | 0.3 ± 0.0 | 0.0 ± 0.0 |
| Al_2_O_3_ | 8.9 ± 0.46 | 8.8 ± 0.45 | 8.9 ± 0.42 | 8.4 ± 0.4 | 8.5 ± 0.4 |
| Fe_2_O_3_ | 0.7 ± 0.09 | 0.7 ± 0.09 | 0.7 ± 0.09 | 0.7 ± 0.1 | 0.8 ± 0.1 |
| MgO | 0.0 ± 0.00 | 0.7 ± 0.12 | 0.2 ± 0.09 | 0.2 ± 0.0 | 0.6 ± 0.1 |
| Cr_2_O_3_ | 0.0 ± 0.00 | 0.0 ± 0.0 | 0.0 ± 0.0 | 0.0 ± 0.0 | 0.0 ± 0.0 |
| Na_2_O | 7.1 ± 0.37 | 7.1 ± 0.5 | 7.1 ± 0.43 | 6.2 ± 0.2 | 6.9 ± 0.4 |
| K_2_O | 3.0 ± 0.18 | 2.7 ± 0.2 | 3.1 ± 0.18 | 3.6 ± 0.1 | 2.9 ± 0.2 |
| P_2_O_5_ | 0.9 ± 0.08 | 0.9 ± 0.1 | 0.8 ± 0.08 | 0.8 ± 0.1 | 0.9 ± 0.1 |
| Total | 100 | 100 | 100 | 100 | 100 |
| OH^-^ concentration (wt.%) | - | 0.145 – 0.275 | - | - | 0.343 – 0.377 |
