# Supplementary material for: Volcanic textures control the mechanical behaviour of Mesoamerican obsidians
Source: R Soc Open Sci. 2026 Oct 14;13(10):261210. doi: 10.1098/rsos.261210 (PMC13682573; doi:10.1098/rsos.261210)
Supplement: Supplementary file 1 [file rsos.261210.zip › rsos.261210/rsos261210_si_003.docx]

**Table S2. Chemical composition (mol%) obtained by EDS area analyses of localized vesicle-filling phases identified in VBO-S and VBO-E for the studied obsidian samples from Sierra de Las Navajas deposit (Hidalgo, Mexico).**

| *Oxides* | *Vesicle / VBO-S* | *Vesicle / VBO-E* |
| --- | --- | --- |
| SiO_2_ | 63.6 ± 1.7 | 67.1 ± 3.3 |
| Al_2_O_3_ | 7.9 ± 0.3 | 8.7 ± 0.5 |
| Fe_2_O_3_ | 1.2 ± 0.1 | 1.0 ± 0.2 |
| MgO | 0.0 ± 0.0 | 0.8 ± 0.2 |
| Cr_2_O_3_ | 4.0 ± 0.2 | 2.1 ± 0.2 |
| Na_2_O | 4.2 ± 0.2 | 7.1 ± 0.5 |
| K_2_O | 12.0 ± 0.1 | 2.9 ± 0.2 |
| P_2_O_5_ | 0.8 ± 0.1 | 0.9 ± 0.2 |
| BaO | 16.3 ± 0.2 | 9.3 ± 1.4 |
| Total | 100 | 100 |
